# Methyl (*R*)-2-(2-chloro­phen­yl)-2-(3-nitro­phenyl­sulfon­yloxy)acetate

**DOI:** 10.1107/S1600536812020016

**Published:** 2012-05-12

**Authors:** Ying-Hua Li, Hong-Wu Xu, Liu-Xue Zhang

**Affiliations:** a Department of Materials and Chemical Engineering, Zhongyuan University of Technology, Zhengzhou, Henan 450007, People’s Republic of China

## Abstract

The reaction between methyl (*R*)-2-(2-chloro­phen­yl)-2-hy­droxy­acetate and 3-nitro­benzene­sulfonyl chloride gave the title compound, C_15_H_12_ClNO_7_S, which is a promising inter­mediate for the synthesis of Clopidrogel, an anti­platelet drug used in the prevention of strokes and heart attacks. In the crystal, mol­ecules are linked through C—H⋯O interactions, and there is also a short Cl⋯O contact present [Cl⋯O = 3.018 (2) Å].

## Related literature

For the synthesis of (*R*)-2-(2-chloro­phen­yl)-2-hy­droxy­acetic acid, see: Bousquet & Musolino (2003 ▶). For related structures, see: Sun *et al.* (2007 ▶); Andersen *et al.* (2007 ▶). For the synthesis of Clopidrogel from sulfonyl­oxyacetic esters of (*R*)-2-(2-chloro­phen­yl)-2-hy­droxy­acetic acid, see: Bousquet & Musolino (1999 ▶); Castaldi *et al.* (2003 ▶); Ema *et al.* (2007 ▶); Zhu *et al.* (2010 ▶). For halogen bonds, see: Bianchi *et al.* (2004 ▶); Fourmigue (2009 ▶); Metrangolo *et al.* (2005 ▶).
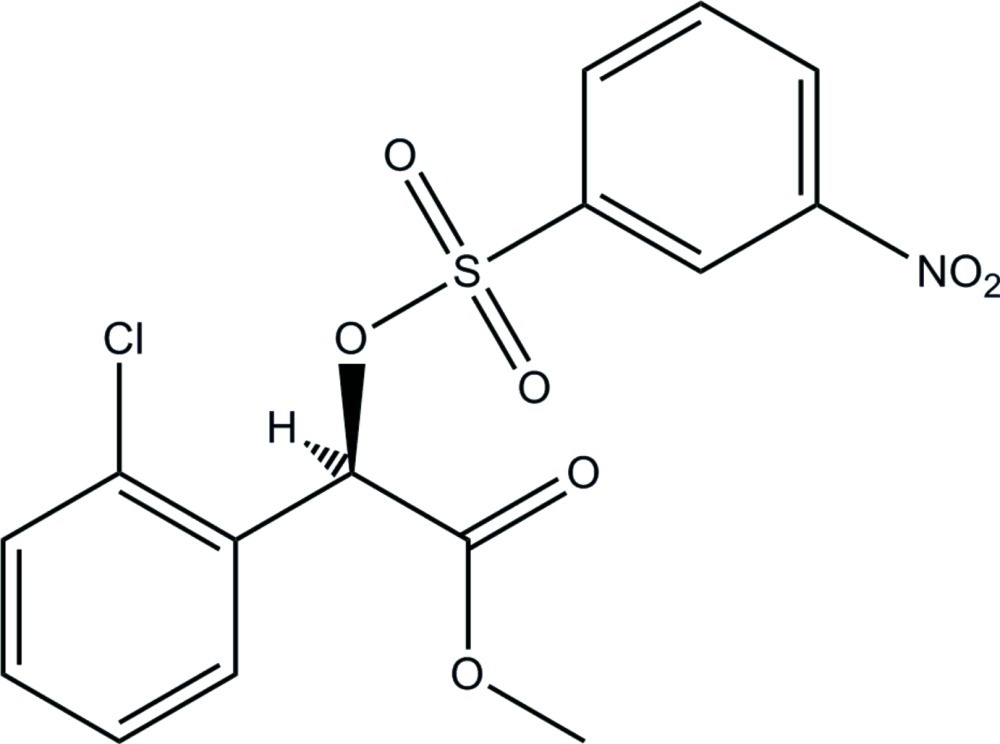


## Experimental

### 

#### Crystal data

C_15_H_12_ClNO_7_S
*M* *_r_* = 385.77
Orthorhombic, 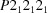

*a* = 7.5791 (3) Å
*b* = 11.0242 (5) Å
*c* = 19.6736 (7) Å
*V* = 1643.80 (11) Å^3^
*Z* = 4
Mo *K*α radiation
μ = 0.40 mm^−1^
*T* = 293 K
0.30 × 0.25 × 0.22 mm

#### Data collection

Agilent Xcalibur Eos Gemini diffractometer
Absorption correction: multi-scan (*Crysalis PRO*; Agilent, 2011 ▶) *T*_min_ = 0.890, *T*_max_ = 0.918
5654 measured reflections
3153 independent reflections
2680 reflections with *I* > 2σ(*I*)
*R*_int_ = 0.023

#### Refinement

*R*[*F* ^2^ > 2σ(*F* ^2^)] = 0.039
*wR*(*F* ^2^) = 0.086
*S* = 1.02
3153 reflections
227 parameters
H-atom parameters constrained
Δρ_max_ = 0.21 e Å^−3^
Δρ_min_ = −0.21 e Å^−3^
Absolute structure: Flack (1983 ▶), 1209 Friedel pairs
Flack parameter: 0.07 (7)

### 

Data collection: *Crysalis PRO* (Agilent, 2011 ▶); cell refinement: *Crysalis PRO*; data reduction: *Crysalis PRO*; program(s) used to solve structure: *SHELXS97* (Sheldrick, 2008 ▶); program(s) used to refine structure: *SHELXL97* (Sheldrick, 2008 ▶); molecular graphics: *SHELXTL/PC* (Sheldrick, 2008 ▶); software used to prepare material for publication: *SHELXTL/PC* and *PLATON* (Spek, 2009 ▶).

## Supplementary Material

Crystal structure: contains datablock(s) I, global. DOI: 10.1107/S1600536812020016/zl2477sup1.cif

Structure factors: contains datablock(s) I. DOI: 10.1107/S1600536812020016/zl2477Isup2.hkl

Supplementary material file. DOI: 10.1107/S1600536812020016/zl2477Isup3.cml

Additional supplementary materials:  crystallographic information; 3D view; checkCIF report

## Figures and Tables

**Table 1:** Hydrogen-bond geometry (Å, °)

*D*—H⋯*A*	*D*—H	H⋯*A*	*D*⋯*A*	*D*—H⋯*A*
C14—H5⋯O4	0.93	2.55	2.920 (4)	104
C14—H5⋯O1^i^	0.93	2.60	3.323 (4)	135
C15—H8*C*⋯O5^ii^	0.96	2.53	3.419 (4)	155

Symmetry codes: (i) 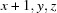; (ii) 
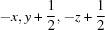
.

**Fig. 1.**
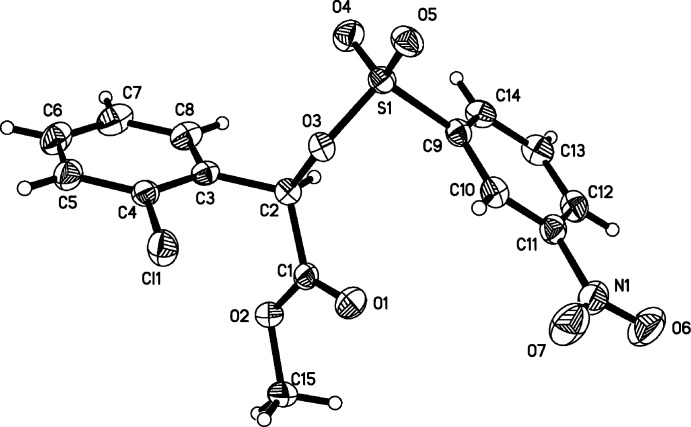
A view of the compound with the atomic numbering scheme. Displacement ellipsoids are drawn at the 30% probability level.

**Fig. 2.**
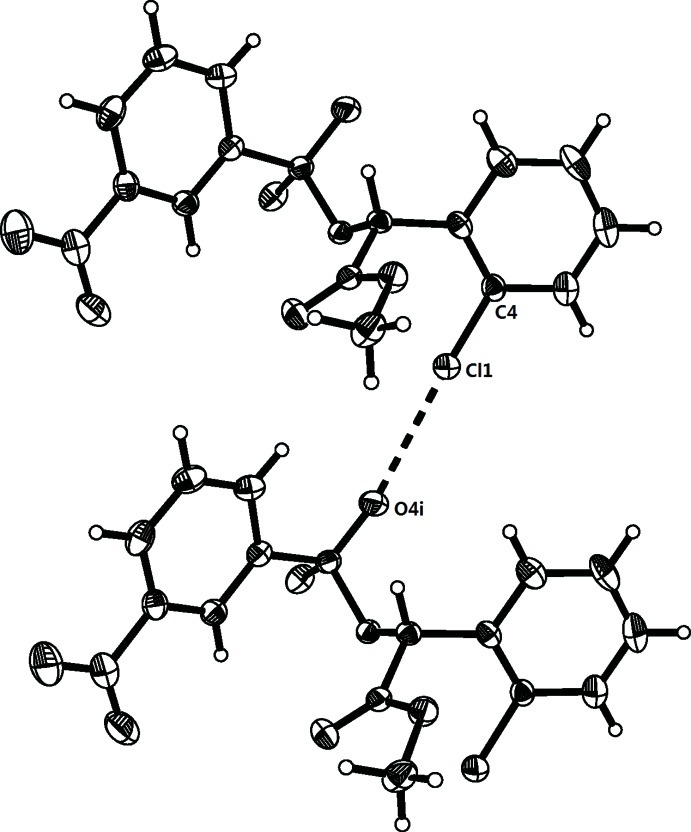
A view of the C—Cl···O interaction (dashed lines) in the crystal structure of the title compound. Symmetry code (i): x - 1, y, z.

**Fig. 3.**
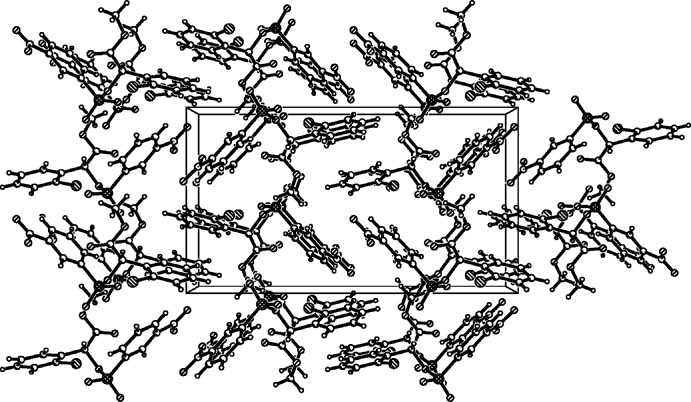
The packing of the compound, viewed down the a axis.

## supplementary crystallographic information

### Comment

Sulfonyloxyacetic esters of (*R*)-methyl-2-(2-chlorophenyl)-2-hydroxyacetate are commonly used in the synthesis of Clopidrogel, an antiplatelet drug used in the prevention of strokes and heart attacks (sold in the United States under the brand name of Plavix) (Bousquet & Musolino, 1999; Castaldi *et al.*, 2003; Ema *et al.*, 2007; Zhu *et al.*, 2010). The title compound, a promising intermediate for the synthesis of Clopidrogel, was obtained in two steps from (*R*)-2-(2-chlorophenyl)-2-hydroxyacetic acid (Bousquet & Musolino, 2003). We report here its crystal structure. In the molecule of the title compound (Fig. 1), the main bond lengths and angles are close to those found in some other derivatives of (*R*)-methyl-2-(2-chlorophenyl)-2-hydroxyacetate (for example, (*R*)-methyl-2-(2-chlorophenyl)-2-(benzenesulfonyloxy) acetate and 4a*R*,11*R*,11*aS*)-11-methyl-9- (trifluoromethyl)-1,2,2,3,4,4a,5,6,11,11adecahydro-pyrido[4,3-*b*] carbazole (*R*)-2-chloromandelate (Sun *et al.*, 2007; Andersen *et al.*, 2007). The crystal structure of this compound is stabilized by an intermolecular halogen bond (Bianchi *et al.*, 2004; Fourmigue, 2009; Metrangolo *et al.*, 2005) between the Cl atom and one of the O atoms of the SO_2_ group of an adjacent molecule, with a C4–Cl1···O4^i^ separation of 3.018 (2) Å (Fig. 2 and Table 1). Symmetry code (i): *x* - 1, *y*, *z*. The crystal structure is also stabilized by intermolecular C–H···O hydrogen bonding interactions (Table 1).

### Experimental

(*R*)-2-(2-Chlorophenyl)-2-hydroxyacetic acid and (*R*)-methyl-2- (2-chlorophenyl)-2-hydroxyacetate were prepared using the established literature procedures (Bousquet *et al.*, 2003, and Sun *et al.*, 2007). A three-necked round-bottomed flask, which was equipped with a magnetic stir bar, was charged with dichloromethane (50 ml), (*R*)-methyl-2- (2-chlorophenyl)-2-hydroxyacetate (4.5 g), triethylamine (4.3 g), and 4,4-dimethylaminopyridine (275 mg). 3-Nitrobenzenesulfonyl chloride (5.5 g) and dichloromethane (50 ml) were added *via* syringe. The mixture was stirred at room temperature for 3 h. The reaction mixture was quenched with water, and washed with 1 N HCl (30 ml) twice. The organic layer was dried over anhydrous sodium sulfate and filtered. After concentration under reduced pressure, the residue was purified by silica gel column chromatography with a mixture of petroleum ether and ethyl acetate (4:1 *v*/*v*) as eluent to give the title compound (yield, 54%). ^1^H NMR (400 MHz, CDCl_3_): 8.648 (s, 1H), 8.432 (d, *J* = 8.0 Hz, 1H), 8.208 (d, *J* = 8.0 Hz, 1H), 7.704 (t, *J* = 8.0 Hz, 1H), 7.376 (d, *J* = 8.0 Hz, 1H), 7.319 - 7.206 (m, 3H), 6.394 (s, 1H), 3.765 (s, 3H) p.p.m.. Well shaped colorless crystals were obtained by slow evaporation of a solution in petroleum ether and ethyl acetate at room temperature for a few days.

### Refinement

All hydrogen atoms were fixed geometrically (C—H bond fixed at 0.93 and 0.96 Å for aromatic and methyl H atoms, respectively) with *U*_iso_(H) = 1.2 (1.5 for methyl groups) times *U*_eq_(C).

### Crystal data

**Table:**

C_15_H_12_ClNO_7_S	*F*(000) = 792
*M**_r_* = 385.77	*D*_x_ = 1.559 Mg m^−^^3^
Orthorhombic, *P*2_1_2_1_2_1_	Mo *K*α radiation, λ = 0.71073 Å
Hall symbol: P 2ac 2ab	Cell parameters from 1814 reflections
*a* = 7.5791 (3) Å	θ = 3.3–26.3°
*b* = 11.0242 (5) Å	µ = 0.40 mm^−^^1^
*c* = 19.6736 (7) Å	*T* = 293 K
*V* = 1643.80 (11) Å^3^	Prism, colourless
*Z* = 4	0.30 × 0.25 × 0.22 mm

### Data collection

**Table:**

Agilent Xcalibur Eos Gemini diffractometer	3153 independent reflections
Radiation source: fine-focus sealed tube	2680 reflections with *I* > 2σ(*I*)
Graphite monochromator	*R*_int_ = 0.023
Detector resolution: 13.6612 pixels mm^-1^	θ_max_ = 26.4°, θ_min_ = 3.3°
ω scans	*h* = −9→9
Absorption correction: multi-scan (*CrysAlis PRO*; Agilent, 2011)	*k* = −13→12
*T*_min_ = 0.890, *T*_max_ = 0.918	*l* = −24→15
5654 measured reflections	

### Refinement

**Table:**

Refinement on *F*^2^	Secondary atom site location: difference Fourier map
Least-squares matrix: full	Hydrogen site location: inferred from neighbouring sites
*R*[*F*^2^ > 2σ(*F*^2^)] = 0.039	H-atom parameters constrained
*wR*(*F*^2^) = 0.086	*w* = 1/[σ^2^(*F*_o_^2^) + (0.0393*P*)^2^ + 0.1718*P*] where *P* = (*F*_o_^2^ + 2*F*_c_^2^)/3
*S* = 1.02	(Δ/σ)_max_ < 0.001
3153 reflections	Δρ_max_ = 0.21 e Å^−^^3^
227 parameters	Δρ_min_ = −0.21 e Å^−^^3^
0 restraints	Absolute structure: Flack (1983), 1209 Friedel pairs
Primary atom site location: structure-invariant direct methods	Flack parameter: 0.07 (7)

### Special details

**Table:**

Geometry. All e.s.d.'s (except the e.s.d. in the dihedral angle between two l.s. planes) are estimated using the full covariance matrix. The cell e.s.d.'s are taken into account individually in the estimation of e.s.d.'s in distances, angles and torsion angles; correlations between e.s.d.'s in cell parameters are only used when they are defined by crystal symmetry. An approximate (isotropic) treatment of cell e.s.d.'s is used for estimating e.s.d.'s involving l.s. planes.
Refinement. Refinement of *F*^2^ against ALL reflections. The weighted *R*-factor *wR* and goodness of fit *S* are based on *F*^2^, conventional *R*-factors *R* are based on *F*, with *F* set to zero for negative *F*^2^. The threshold expression of *F*^2^ > σ(*F*^2^) is used only for calculating *R*-factors(gt) *etc*. and is not relevant to the choice of reflections for refinement. *R*-factors based on *F*^2^ are statistically about twice as large as those based on *F*, and *R*- factors based on ALL data will be even larger.

### Fractional atomic coordinates and isotropic or equivalent isotropic displacement parameters (Å^2^)

**Table:**

	*x*	*y*	*z*	*U*_iso_*/*U*_eq_	
S1	0.47477 (9)	0.03812 (6)	0.23919 (4)	0.03772 (18)	
Cl1	−0.05655 (10)	0.08212 (8)	0.36889 (4)	0.0513 (2)	
N1	0.2766 (4)	0.3236 (3)	0.04654 (14)	0.0554 (7)	
O7	0.1312 (4)	0.2781 (3)	0.05356 (13)	0.0763 (8)	
O6	0.3113 (4)	0.4007 (2)	0.00436 (14)	0.0820 (8)	
O5	0.4263 (3)	−0.06820 (17)	0.20270 (10)	0.0474 (5)	
O4	0.6140 (3)	0.0334 (2)	0.28718 (11)	0.0540 (6)	
O2	0.1443 (3)	0.36269 (18)	0.33486 (10)	0.0449 (5)	
O1	0.0857 (3)	0.2602 (2)	0.23895 (10)	0.0544 (6)	
O3	0.2979 (2)	0.07757 (16)	0.27557 (9)	0.0342 (4)	
C10	0.3826 (4)	0.1918 (3)	0.13767 (14)	0.0400 (7)	
H1A	0.2728	0.1541	0.1383	0.048*	
C11	0.4194 (4)	0.2837 (3)	0.09275 (14)	0.0417 (7)	
C12	0.5830 (4)	0.3385 (3)	0.08930 (16)	0.0496 (8)	
H3	0.6055	0.3984	0.0571	0.060*	
C13	0.7118 (4)	0.3032 (3)	0.13422 (17)	0.0504 (8)	
H4	0.8222	0.3399	0.1327	0.061*	
C14	0.6791 (4)	0.2137 (3)	0.18141 (16)	0.0442 (7)	
H5	0.7656	0.1913	0.2125	0.053*	
C9	0.5149 (4)	0.1576 (2)	0.18190 (13)	0.0369 (6)	
C1	0.1619 (4)	0.2713 (3)	0.29162 (14)	0.0371 (6)	
C15	0.0212 (4)	0.4558 (3)	0.31462 (17)	0.0591 (9)	
H8A	0.0540	0.4868	0.2708	0.089*	
H8B	0.0231	0.5204	0.3473	0.089*	
H8C	−0.0954	0.4221	0.3123	0.089*	
C2	0.3017 (3)	0.1846 (2)	0.31890 (13)	0.0336 (6)	
H9	0.4171	0.2235	0.3133	0.040*	
C3	0.2823 (4)	0.1498 (2)	0.39238 (14)	0.0352 (6)	
C8	0.4258 (4)	0.1657 (3)	0.43647 (15)	0.0473 (7)	
H11	0.5314	0.1963	0.4196	0.057*	
C7	0.4125 (5)	0.1367 (3)	0.50432 (17)	0.0584 (9)	
H12	0.5084	0.1482	0.5331	0.070*	
C6	0.2564 (5)	0.0905 (3)	0.52935 (15)	0.0588 (10)	
H13	0.2472	0.0717	0.5753	0.071*	
C5	0.1147 (5)	0.0719 (3)	0.48751 (15)	0.0495 (8)	
H14	0.0108	0.0391	0.5046	0.059*	
C4	0.1279 (4)	0.1026 (3)	0.41929 (13)	0.0378 (7)	

### Atomic displacement parameters (Å^2^)

**Table:**

	*U*^11^	*U*^22^	*U*^33^	*U*^12^	*U*^13^	*U*^23^
S1	0.0318 (3)	0.0408 (4)	0.0406 (4)	0.0007 (3)	0.0028 (3)	0.0023 (3)
Cl1	0.0389 (4)	0.0714 (5)	0.0437 (4)	−0.0063 (4)	−0.0013 (3)	0.0053 (4)
N1	0.069 (2)	0.0531 (17)	0.0436 (15)	0.0037 (16)	−0.0046 (15)	0.0024 (14)
O7	0.0629 (16)	0.100 (2)	0.0655 (16)	−0.0087 (15)	−0.0233 (14)	0.0133 (16)
O6	0.104 (2)	0.0721 (18)	0.0705 (16)	0.0062 (17)	−0.0065 (17)	0.0295 (16)
O5	0.0533 (13)	0.0350 (11)	0.0539 (12)	0.0010 (9)	0.0129 (11)	−0.0059 (9)
O4	0.0383 (11)	0.0687 (14)	0.0548 (13)	0.0030 (11)	−0.0034 (10)	0.0141 (12)
O2	0.0473 (12)	0.0420 (11)	0.0456 (11)	0.0095 (9)	0.0025 (10)	0.0033 (10)
O1	0.0526 (13)	0.0699 (15)	0.0407 (11)	0.0089 (11)	−0.0095 (11)	0.0048 (11)
O3	0.0314 (9)	0.0376 (10)	0.0338 (9)	−0.0052 (8)	0.0008 (8)	−0.0048 (8)
C10	0.0378 (15)	0.0438 (17)	0.0384 (15)	−0.0057 (13)	−0.0006 (13)	−0.0064 (14)
C11	0.0507 (18)	0.0399 (16)	0.0345 (14)	0.0000 (14)	−0.0008 (14)	−0.0044 (13)
C12	0.064 (2)	0.0359 (16)	0.0491 (18)	−0.0059 (15)	0.0150 (18)	0.0029 (14)
C13	0.0412 (17)	0.0444 (18)	0.066 (2)	−0.0113 (14)	0.0076 (17)	−0.0015 (17)
C14	0.0352 (16)	0.0423 (16)	0.0551 (18)	−0.0029 (13)	0.0016 (15)	−0.0037 (15)
C9	0.0364 (15)	0.0370 (14)	0.0373 (14)	−0.0026 (12)	0.0047 (13)	−0.0016 (12)
C1	0.0303 (15)	0.0449 (16)	0.0361 (14)	−0.0003 (12)	0.0022 (13)	0.0069 (14)
C15	0.056 (2)	0.056 (2)	0.066 (2)	0.0220 (17)	0.0167 (18)	0.0188 (18)
C2	0.0305 (14)	0.0344 (14)	0.0360 (14)	−0.0042 (12)	−0.0024 (13)	−0.0030 (12)
C3	0.0426 (15)	0.0288 (14)	0.0342 (14)	0.0039 (12)	−0.0077 (13)	−0.0058 (12)
C8	0.0567 (19)	0.0361 (16)	0.0493 (17)	−0.0013 (14)	−0.0122 (17)	−0.0049 (14)
C7	0.080 (3)	0.0479 (19)	0.0473 (18)	0.0004 (19)	−0.0303 (19)	−0.0086 (16)
C6	0.094 (3)	0.0486 (19)	0.0340 (16)	0.0087 (19)	−0.0082 (19)	−0.0031 (16)
C5	0.066 (2)	0.0449 (18)	0.0378 (15)	0.0056 (16)	0.0015 (15)	0.0010 (14)
C4	0.0425 (16)	0.0377 (15)	0.0332 (14)	0.0062 (12)	−0.0016 (13)	−0.0024 (13)

### Geometric parameters (Å, º)

**Table:**

S1—O4	1.417 (2)	C13—H4	0.9300
S1—O4	1.417 (2)	C14—C9	1.390 (4)
S1—O5	1.423 (2)	C14—H5	0.9300
S1—O3	1.5809 (18)	C1—C2	1.524 (4)
S1—C9	1.760 (3)	C15—H8A	0.9600
Cl1—C4	1.728 (3)	C15—H8B	0.9600
N1—O6	1.217 (3)	C15—H8C	0.9600
N1—O7	1.218 (4)	C2—C3	1.503 (4)
N1—C11	1.480 (4)	C2—H9	0.9800
O2—C1	1.326 (3)	C3—C4	1.386 (4)
O2—C15	1.443 (3)	C3—C8	1.402 (4)
O1—C1	1.192 (3)	C8—C7	1.376 (4)
O3—C2	1.456 (3)	C8—H11	0.9300
C10—C11	1.373 (4)	C7—C6	1.379 (5)
C10—C9	1.380 (4)	C7—H12	0.9300
C10—H1A	0.9300	C6—C5	1.368 (4)
C11—C12	1.381 (4)	C6—H13	0.9300
C12—C13	1.374 (4)	C5—C4	1.388 (4)
C12—H3	0.9300	C5—H14	0.9300
C13—C14	1.377 (4)		
			
O4—S1—O5	119.91 (14)	O1—C1—C2	125.3 (3)
O4—S1—O5	119.91 (14)	O2—C1—C2	108.7 (2)
O4—S1—O3	109.87 (11)	O2—C15—H8A	109.5
O4—S1—O3	109.87 (11)	O2—C15—H8B	109.5
O5—S1—O3	103.66 (11)	H8A—C15—H8B	109.5
O4—S1—C9	108.98 (13)	O2—C15—H8C	109.5
O4—S1—C9	108.98 (13)	H8A—C15—H8C	109.5
O5—S1—C9	109.75 (12)	H8B—C15—H8C	109.5
O3—S1—C9	103.33 (12)	O3—C2—C3	110.7 (2)
O6—N1—O7	124.1 (3)	O3—C2—C1	106.7 (2)
O6—N1—C11	117.9 (3)	C3—C2—C1	115.5 (2)
O7—N1—C11	118.0 (3)	O3—C2—H9	107.9
C1—O2—C15	115.4 (2)	C3—C2—H9	107.9
C2—O3—S1	118.10 (15)	C1—C2—H9	107.9
C11—C10—C9	117.4 (3)	C4—C3—C8	117.7 (3)
C11—C10—H1A	121.3	C4—C3—C2	123.1 (2)
C9—C10—H1A	121.3	C8—C3—C2	119.2 (3)
C10—C11—C12	122.5 (3)	C7—C8—C3	121.0 (3)
C10—C11—N1	117.7 (3)	C7—C8—H11	119.5
C12—C11—N1	119.8 (3)	C3—C8—H11	119.5
C13—C12—C11	118.8 (3)	C8—C7—C6	119.6 (3)
C13—C12—H3	120.6	C8—C7—H12	120.2
C11—C12—H3	120.6	C6—C7—H12	120.2
C12—C13—C14	120.6 (3)	C5—C6—C7	120.9 (3)
C12—C13—H4	119.7	C5—C6—H13	119.5
C14—C13—H4	119.7	C7—C6—H13	119.5
C13—C14—C9	119.0 (3)	C6—C5—C4	119.3 (3)
C13—C14—H5	120.5	C6—C5—H14	120.4
C9—C14—H5	120.5	C4—C5—H14	120.4
C10—C9—C14	121.6 (3)	C3—C4—C5	121.4 (3)
C10—C9—S1	118.9 (2)	C3—C4—Cl1	120.9 (2)
C14—C9—S1	119.5 (2)	C5—C4—Cl1	117.7 (2)
O1—C1—O2	125.9 (3)		

### Hydrogen-bond geometry (Å, º)

**Table:**

*D*—H···*A*	*D*—H	H···*A*	*D*···*A*	*D*—H···*A*
C14—H5···O4	0.93	2.55	2.920 (4)	104
C14—H5···O1^i^	0.93	2.60	3.323 (4)	135
C15—H8*C*···O5^ii^	0.96	2.53	3.419 (4)	155
C4—Cl1···O4^iii^	1.73 (1)	3.02 (1)	4.744 (4)	176 (1)

Symmetry codes: (i) *x*+1, *y*, *z*; (ii) −*x*, *y*+1/2, −*z*+1/2; (iii) *x*−1, *y*, *z*.
